# The accuracy of cortical bone trajectory screw placement guided by spinous process clamp hardware in lumbar spinal surgery: a retrospective study

**DOI:** 10.1038/s41598-023-43406-1

**Published:** 2023-09-30

**Authors:** Xi-nuo Zhang, Qing-jun Su, Bao-qing Pei, Ai-xing Pan, Hong-hao Yang, Hong-tao Ding, Yong Hai, Yu-zeng Liu

**Affiliations:** 1 Department of Orthopedic Surgery, Beijing Chao-Yang Hospital, Capital Medical University, 8 Gong Ti Nan Lu, Chaoyang District, Beijing, 100020 China; 2 Beijing Key Laboratory for Design and Evaluation Technology of Advanced Implantable and Interventional Medical Devices, Beijing Advanced Innovation Center for Biomedical Engineering, School of Biological Science and Medical Engineering, Beihang University, Beijing, 100083 China

**Keywords:** Medical research, Musculoskeletal system

## Abstract

This study aimed to assess the accuracy of cortical bone trajectory (CBT) screws placement guided by a spinous process clamp (SPC) guide. A total of 32 patients who received single-level midline lumbar fusion (MIDLF) surgery between June 2019 and January 2020 were retrospectively analyzed and divided into free-hand (FH) and SPC-guided groups according to the surgical approach. In the FH group, CBT screws was implanted with the assistance of fluoroscopy, while in the SPC group, CBT screws was implanted using the SPC navigator hardwire. A total of 128 screws were assessed in this study, with higher rates of clinically acceptable screw placement (grades A and B) and grade A screws in the SPC group than in the FH guide group (92.2% vs. 79.7%, *P* = 0.042 and 54.7% vs. 35.9%, *P* = 0.033, respectively). Misplacement screws (grades C, D, and E) occurred more often in the FH group than in the SPC guide group (20.3% vs. 7.8%, *P* = 0.042). The incidence of proximal facet joint violation (FJV) was higher in the FH group than in the SPC group (15.6% vs. 3.1%, *P* = 0.030). The radiation dose and time in the SPC guide group were comparable to those in the FH group (*P* = 0.063 and *P* = 0.078). The average operative time was significantly longer in the SPC guide group than in the FH group (267.8 ± 45.5 min vs. 210.9 ± 44.5 min, *P* = 0.001). Other clinical parameters, such as the average bone mineral density (BMD), intraoperative blood loss, and postoperative hospital stay, were not significantly different. Oswestry disability index (ODI) and back pain visual analogue scale (VAS) scores were significantly improved in both groups compared with preoperatively. SPC guided screw placement was more accurate than the fluoroscopy-assisted FH technique for single-level MIDLF at L4/5. Patients undergoing SPC-guided screw placement can achieve similar clinical outcomes as the fluoroscopy-assisted FH technique.

## Introduction

Posterior lumbar interbody fusion (PLIF) is an effective therapy for lumbar degenerative diseases and has been widely used in spinal surgery^1^. However, pedicle screw implantation can be problematic in patients with osteoporosis and obesity^2^. In addition, the starting point for pedicle screws (PS) is lateral to the midline, near the lateral wall of the facet joint, resulting in the risk of a facet joint violation (FJV) during dissection of the paravertebral muscles or screw placement^3,4^. In 2009, Santoni et al.^5^ first reported the cortical bone trajectory (CBT) screw fixation technique, which compensated for the biomechanical deficiency of pedicle screw fixation in patients with osteoporosis. Compared with PS, the CBT method maximizes the purchase of the pedicle and vertebral cortical bone through the screw thread contact with the cortical bone and enhances the fixation strength. Lumbar fixation with CBT technology has been described as a midline lumbar fusion (MIDLF) surgery^6^. The new pathway follows a caudo-cephalad sagittal path and a medial–lateral path in the axial plane, which reduces the skin incision length, muscle dissection, and superior facet joint exposure.

Previous studies have reported screw misalignment rates between 0 and 12.5%^7–9^. Three-dimensional (3D) printed template guides, 3D navigation, and robotic-assisted surgery systems have been developed to improve accuracy of screw placement^10–13^. However, an extended paraspinal soft tissue dissection and increased radiation exposure limit their widespread application^14^. Based on the experience of CBT surgery and the advantages and disadvantages of existing navigators, our center has developed and designed 3D spinal navigation technology with independent intellectual property rights. This 3D spine navigation technique relies on bone-anchored spine tracking guided by spinous process clips (SPC). In this study, we will compare the accuracy of screw placement using SPC technique with fluoroscopy-assisted free-hand (FH) technique and introduce the SPC technique by means of a retrospective study.

## Materials and methods

### Patients

A review of patients who were diagnosed with lumbar degenerative diseases and underwent single-level MIDLF surgery at L4/5 with CBT screw internal fixation from June 2019 to January 2020, identified a total of 32 patients who were eligible. Patients were divided into two groups according to surgical approach: FH (16 cases) and SPC guide (16 cases) groups. All surgeries were performed by the same surgeon. This study was approved by the institutional review board.

The inclusion criteria were as follows: (1) a diagnosis of L4/5 single-segment lumbar degenerative diseases, (2) ineffective conservative treatment for > 6 months, (3) follow-up time ≥ 1 years, and (4) complete preoperative and postoperative imaging data. Exclusion criteria included the following: (1) a history of lumbar surgery; (2) severe neurological symptoms (incomplete paralysis, cauda equina syndrome, etc.); (3) spinal deformity with Cobb angle more than 20°; (4) spinal tumor; (5) severe osteoporosis (a T-score determined by the dual-energy X-ray absorptiometry (DXA) method ≤ − 2.5, associated with osteoporotic fracture); (6) Lumbar fracture and (7) comorbidities with contraindications to surgery (such as Parkinson's disease, mental illness) with similar diseases and systemic conditions that cannot tolerate surgery.

### Preoperative planning

**Figure 1:**
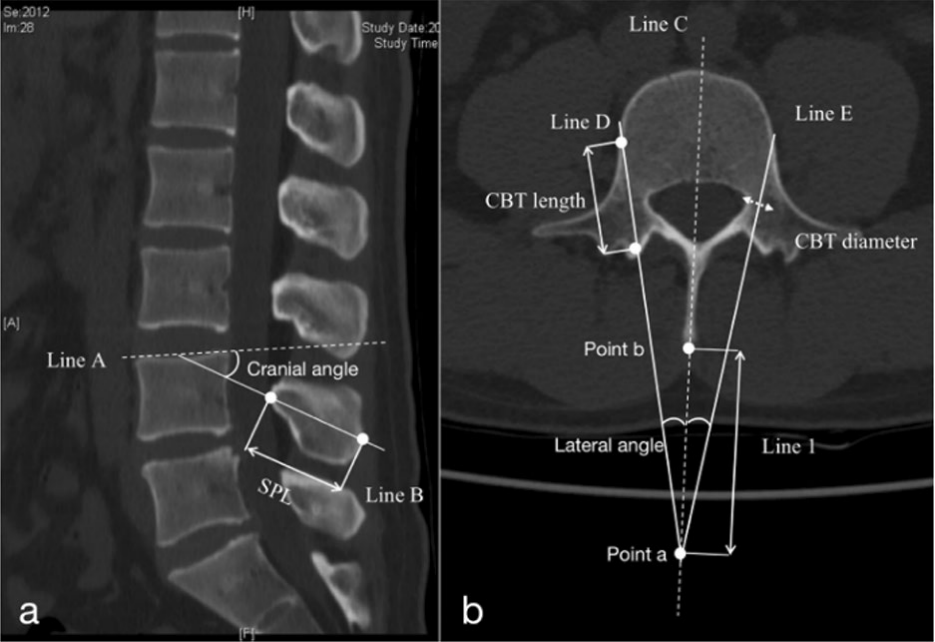
**(a**) the CBT screws and SPC guide parameters in a sagittal image; (**b**): the CBT screws and SPC guide parameters in an axial image.

The 3D CT scans of the lumbar spine were generated using Picture Archiving and Communication Software (PACS) (General Electric Medical Systems, Milwaukee, WI, USA). The 3D reconstructions were used to plan the caudocephalad and medial–lateral paths of CBT screws (Fig. 1). In the sagittal plane, line A passed through the upper endplate of the vertebra, line B was axial to the CBT screw, and the angle between lines A and B was the cranial angle. The spinous process length (SPL) was the maximum length of the spinous process. In the axial plane, line C represented the symmetry line of the vertebra and the spinous process, and lines D and E represented the axes of the CBT screw on both sides. For the SPC guide, the three lines intersected at a point where the guide knob was located. The distance between the knob and the spinous process was line 1. The angle between lines D/E to line C was the lateral angle. The screw length was the length of lines D/E inside the vertebra. The screw radius was the shortest distance between the screw axis and pedicle wall.

### Spinous process clamp (SPC)-assisted CBT screws implantation

**Figure 2:**
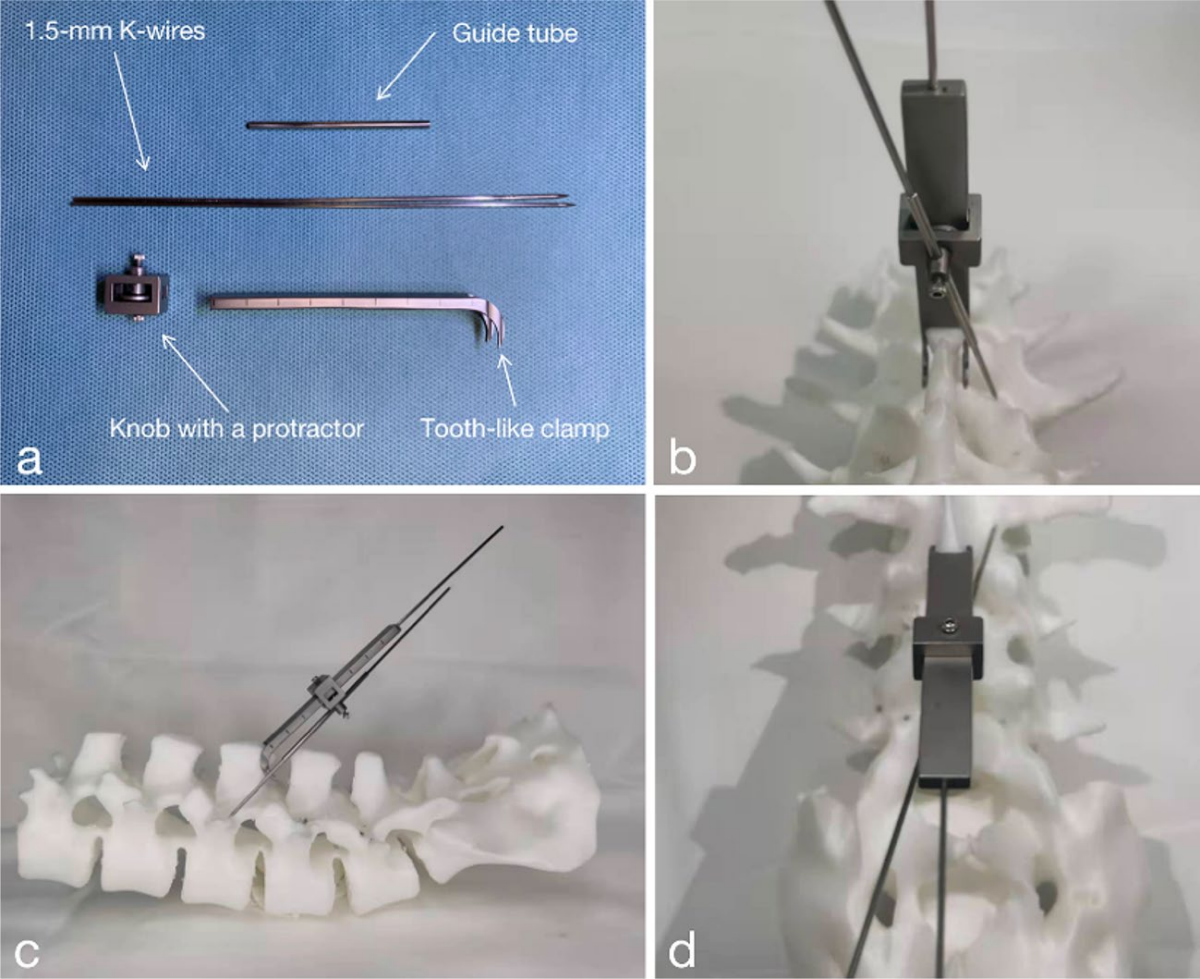
(**a**) Hardware of the SPC guide; (**b**) the SPC guide was anchored on the specimen in cranial view; (**c**) the SPC guide was anchored on the specimen in sagittal view; (**d**) the SPC guide was anchored on the specimen in caudal view.

**Figure 3:**
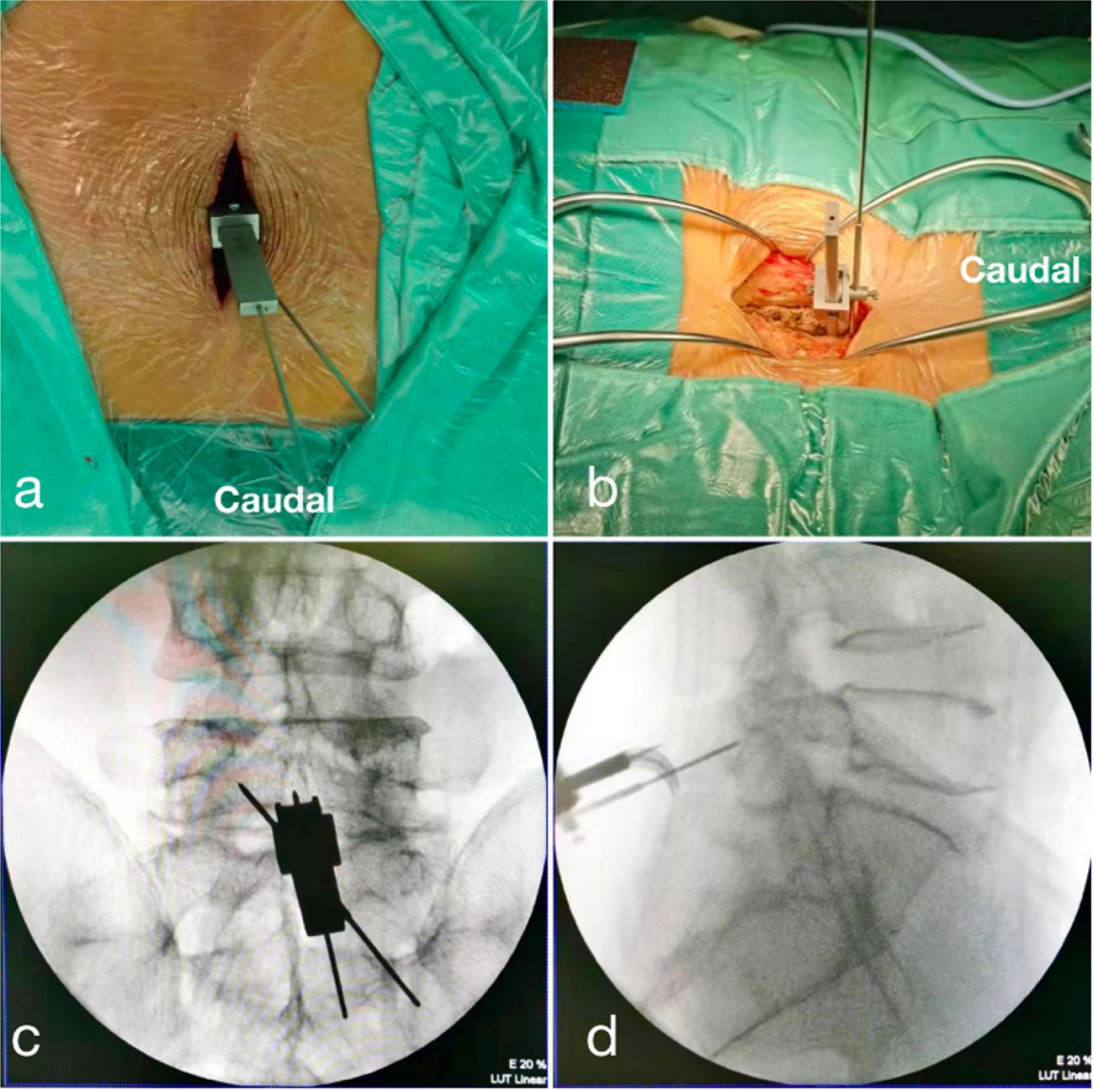
(**a**) Looking down at the hardware of the SPC guide during the surgery; (**b**) the SPC guide was anchored on the spinous process during the surgery in sagittal view; (**c**) intraoperative C-arm coronal view of the SPC guide; (**d**) intraoperative C-arm lateral view of the SPC guide.

Preoperative measurements should be made in sagittal and coronal CT images of the lumbar spine (Fig. 1). The SPC guide consisted of a spinous process clamp, column, knob with a protractor, guide tube, and two 1.5-mm Kirschner wires (K-wires) (Fig. 2). SPC guides are securely attached to the spinous process of the operative vertebral body with special curved toothed clips (Fig. 3a,b). The SPC guide with a bilateral clamp anchored the device on the caudal part of the spinous process during the procedure. After the SPC guide was anchored at the spinous process, according to lateral fluoroscopy image, the column was adjusted to the pre-measured caudo-cephalad angle (Cranial angle in Fig. 1a) and locked by 1.5-mm K-wires through the column (Fig. 3c,d). The knob with protractor was locked at the height based on preoperative measurement. According to preoperative measured lateral angle from CT, the lateral angle of the guide tube was adjusted (Lateral angle in Fig. 1b). The second 1.5 mm K-wire was inserted through the guide tube that anchored to the surface of pars interarticularis with an electric drill. Coronal imaging confirmed the starting point of the cortical bone trajectory at the inner lower edge of the pedicle projection. The pedicle was tapped by hollow screw taps along the K-wire, and the hollow CBT screws were implanted through the K-wire. To minimize the pedicle perforation area, the preoperative CBT screw planning recommended a 4.5-mm diameter CBT screw when the pedicle was too narrow to be inserted.

### Fluoroscopy-assisted free-hand (FH) CBT screws implantation

Fluoroscopy-assisted Free-hand CBT screw placement followed the standard MIDLF surgical approach. The standard MIDLF surgical incision was 1–2 cm smaller than the pedicle screw incision because CBT instruments were placed more caudally than PS instruments^15^. The cranial exposure stopped at the inferior aspect of the facet joint, and the lateral exposure stopped at the lateral border of the pars region. The exact entry point was the intersection of the lateral margin of the pars interarticularis and the inferior level of the transverse process^16^. A 1.7-mm drill bit was used to drill the complete path from the entry point along the cortical bone trajectory, approximately 10° laterally and 30° cranially, and stopped at a depth of 20–25 mm. A burr was used to probe the tactile feedback through the pedicle wall before the CBT screw was inserted along the trajectory. With the assistance of lateral and anteroposterior fluoroscopy and identification of anatomical landmarks, bilateral CBT instruments of the cortical screw fixation system (Medtronic CD Solera 4.75 spinal system) were implanted at the appropriate position^16^.

### Assessment

Postoperative CT reconstruction was used to assess the accuracy of the screw placement between the SPC guide and FH groups. The location of the screw inside the vertebral bodies or pedicle was measured in millimeters in the medial, lateral, cranial, caudal, and cephalad endplate and vertebral cortex directions^17^. The screw accuracy was graded based on the modified Gertzbein–Robbins method (grade A, no perforation; grade B, 0–2 mm; grade C, 2–4 mm; grade D, 4–6 mm; and grade E > 6 mm)^2,18^. Perforations worse than grade C were deemed unacceptable^11^. The assessment of the proximal FJV included adjacent cranial and all fixed segments^19^. The cranial and lateral angles of the CBT screws were assessed in the sagittal and axial planes, respectively. Radiation exposure parameters were obtained from the C-arm equipment. Clinical data, such as Oswestry disability index (ODI), and back pain visual analog scale (VAS) scores, were evaluated preoperatively and at final follow-up.

### Statistical analysis

Statistical analyses were performed using SPSS, version 25.0 (IBM Corp., Armonk, New York, USA). Continuous variables are presented as mean ± standard deviation (SD), whereas non-continuous data are presented as numbers or ratios. For comparison between groups, the χ2 test was used for categorical variables (if the frequency was less than 5, Fisher's exact test was used); non-continuous data were compared using Pearson's chi-square test. Statistical significance was considered as *P* < 0.05.

### Ethics approval and consent to participate

The study complied with the Declaration of Helsinki and was approved by the Ethics Committee of the Beijing Chao-yang Hospital, Capital Medical University. Informed consent was waived because of the retrospective study design, which was approved by Ethics Committee of Beijing Chao-Yang Hospital, Capital Medical University.

## Results

### Baseline characteristics

A total of 32 patients in two groups including 12 (37.5%) males and 20 (62.5%) females, with a mean age of 61.9 ± 5.8 years (range: 54–72 years), follow-up time of 15.0 ± 2.1 months (range: 12–21 months), and body mass index (BMI) of 23.9 ± 3.8 kg/m^2^ (15.00 ~ 32.00 kg/m^2^). The operative time in the SPC guide group was significantly longer than that in the FH group (267.8 ± 45.5 min vs. 210.9 ± 44.5 min, *P* = 0.001). The BMD, intraoperative blood loss, and length of postoperative hospital stay were not significantly different between the groups (Table 1).

**Table 1:** Patient demographics.

Variables	Total (n = 32)	FH group (n = 16)	SPC guide group (n = 16)	T value	*P* value
Age (y/o)	61.9 ± 5.8	62.2 ± 5.8	61.6 ± 5.9	0.272	0.788
Sex^#^ (M, %)	12 (37.5%)	5 (31.1%)	7 (43.8%)	–	0.716
Follow-up (m)	15.0 ± 2.1	14.9 ± 1.6	15.2 ± 2.6	− 0.412	0.683
BMI (kg/m2)	23.9 ± 3.8	24.1 ± 4.0	23.8 ± 3.6	0.184	0.855
BMD T-value	− 1.3 ± 1.8	− 1.5 ± 1.7	− 1.0 ± 1.9	− 0.822	0.418
Operative time (min)	239.4 ± 52.9	210.9 ± 44.5	267.8 ± 45.5	− 3.573	0.001*
Bleeding (ml)	338.4 ± 159.2	372.52 ± 169.2	304.4 ± 145.8	1.220	0.232
LOS (d)	11.8 ± 3.3	12.3 ± 3.7	11.3 ± 2.9	0.903	0.374
Radiation dose (μSv)	81.8 ± 14.3	73.8 ± 8.6	89.7 ± 14.6	0.031	0.078
Radiation time (s)	80.3 ± 12.2	74.0 ± 6.4	86.7 ± 13.4	0.011	0.063
Preoperative ODI	62.8 ± 2.6	63.3 ± 3.2	62.2 ± 1.9	1.215	0.234
ODI at last follow-up	12.1 ± 2.2	11.7 ± 1.5	12.5 ± 2.6	− 1.066	0.295
Preoperative back pain VAS	6.8 ± 0.7	6.9 ± 0.6	6.7 ± 0.8	0.241	0.462
Back pain VAS at last follow-up	1.7 ± 0.8	1.8 ± 0.8	1.6 ± 0.8	− 0.214	0.658

FH, Freehand; SPC, Spinous process clamp; y/o, years old; BMI, Body mass index; BMD T-value, bone mineral density T-value; min, minute; ml, millimeter; d, day; μSv, microsievert; s, second; M, Male; SPC, Spinous Process Clamp guide. Lumbar segments varied from the first lumbar to the first sacral segment. BMD was measured using dual energy X-ray absorptiometry (DXA).

*P < 0.05, as compared with the two groups value.

^#^The total number of cases in this study was < 40 or theoretical frequency < 1, so the results of Fisher's Exact Test were used.

### Screw accuracy outcome

Table 2 shows the screw accuracy grades. A total of 128 CBT screws were inserted. Overall, the proportion of clinically acceptable screw placements (corresponding to grades A and B) was higher in the SPC than in the FH group (92.2% vs. 79.7%, *P* = 0.042). The proportion of remaining screws (grades C, B, and E) was greater in the FH than in the SPC group (20.3% vs. 7.8%, *P* = 0.042).

**Table 2:** Screw placement quality with the modified Gertzbein–Robbins classification.

Grade	Total (n = 128)	FH group (n = 64)	SPC guide group (n = 64)	χ^2^/T-value	*P* value
A (n %)	58 (45.3%)	23 (35.9%)	35 (54.7%)	4.540	0.033*
B (n %)	52 (40.6%)	28 (40.6%)	24 (37.5%)	0.518	0.472
A + B (n %)	110 (84.9%)	51 (79.7%)	59 (92.2%)	4.137	0.042*
C (n %)	11 (8.6%)	7 (10.9%)	4 (6.3%)	0.895	0.344
D^#^ (n %)	5 (3.9%)	4 (6.3%)	1 (1.6%)	–	0.365
E^#^ (n %)	2 (3.1%)	2 (3.1%)	0 (0.0%)	–	0.154
C + D + E (n %)	18 (14.1%)	13 (20.3%)	5 (7.8%)	4.137	0.042*
FJV (n %)	12 (9.4%)	10 (15.6%)	2 (3.1%)	4.506	0.034*
Lateral angle (°)	8.5 ± 3.2	6.4 ± 3.1	10.1 ± 2.0	− 3.972	> 0.001*
Cranial angle (°)	25.3 ± 5.5	23.0 ± 4.4	27.7 ± 5.5	− 2.716	0.011*

FJV, Facet joint violation, NA, Not available. Values are expressed as the mean ± standard deviation or number.

*P < 0.05, as compared with the two groups value.

^#^The total number of cases in this study was < 40 or theoretical frequency < 1, so the results of Fisher's Exact Test were used.

**Figure 4:**
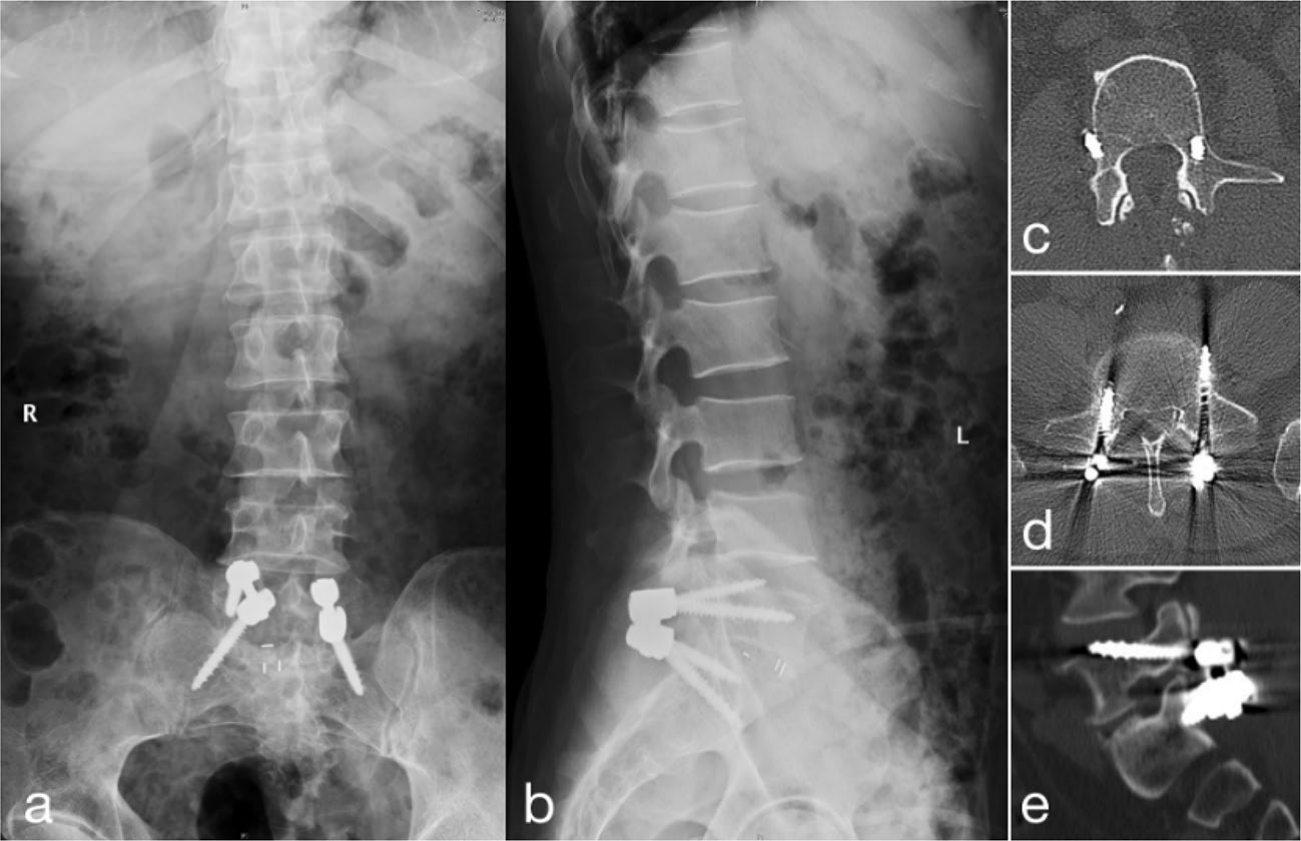
(**a**) Postoperative anterio–posterior imaging of the SPC guide assisted CBT screw placement; (**b**) Postoperative lateral imaging of the SPC guide assisted CBT screw placement; (**c**) CT axial imaging of lateral pedicle perforation in the FH group; (**d**) CT axial imaging of vertebral cortex perforation in FH group; (**e**) CT sagittal imaging of cephalad endplate perforation in the SPC group.

The direction of screw misplacement is presented in Table 3. In the FH group, the most common direction of screw misplacement was vertebral cortex displacement (four screws), followed by caudal pedicle displacement (three screws). In the SPC guide group, a vertebral cephalad endplate perforation was the most common (two screws). Figure 4 shows postoperative X-ray and CT scans used to evaluate the CBT screw accuracy. No patient presented with neurological symptoms due to screw malposition. None of the patients with screw malposition underwent revision surgery after close postoperative follow-up and evaluation.

**Table 3:** Screw deviations with grades (C, D, and E).

Deviation	FH group (n = 13)	SPC guide group (n = 5)
Cranial pedicle n (%)	1 (7.69%)	0 (0%)
Caudal pedicle n (%)	3 (23.08)	1 (20.00%)
Cephalad endplate n (%)	1 (7.69%)	2 (40.00%)
Medial pedicle n (%)	2 (15.38%)	0 (0%)
Lateral pedicle n (%)	2 (15.38%)	1 (20.00%)
Vertebral cortex n (%)	4 (30.77%)	1 (20.00%)

The incidence of proximal FJV in the SPC guide group was significantly lower than that in the FH group (3.1% vs. 15.6%, *P* = 0.030). The cranial angle of the cortical screw in the SPC guide group was much larger than that in the FH group (27.7 ± 5.5° vs. 23.0 ± 4.4°, *P* = 0.011), as was the lateral angle of the cortical screw (10.1 ± 2.0° vs. 6.4 ± 3.1°, *P* > 0.001) (Table 2).

### Secondary outcomes

The radiation exposure time and cumulative radiation dose of exposure to the staff in the SPC guide group was comparable to that in the FH group (*P* = 0.063 and *P* = 0.078, respectively). There were no significant differences in ODI and back pain VAS scores in the preoperative and final follow-up periods (≥ 12 months postoperatively) between both groups (Table 1).

## Discussion

Various tools have been developed such as orthopedic surgical robotics, 3D printed navigation guidance to improve accuracy and safety of screw placement^2,11,12^. Hussain et al.^12^ used 3D navigation-guided cortical bone trajectory screws for lumbar spondylolisthesis. The patient showed significant improvement after surgery. Li et al.^13^ demonstrated that robot-assisted techniques can improve accuracy of screw placement while reducing radiation exposure to the medical team. However, the advantages of these techniques over FH fluoroscopy-assisted techniques have been controversial. In our center, CBT screw placement assisted by SPC showed a higher accuracy of screw placement and a lower incidence of FJV compared with fluoroscopy-assisted Free-Hand screw placement.

### Accuracy and safety

To our knowledge, no similar studies have reported the accuracy and safety of CBT screw placement using fluoroscopy-assisted FH versus SPC guides. Our results show that the accuracy of screws in clinically acceptable positions in the SPC guide group was significantly higher than that in the FH group. Because the SPC directly joins the spinous process to constitute a hard tissue connection, SPC guidelines based on preoperative planning provide accurate and effective screw placement. The following precautions should be taken when using this technique in surgery. Accurate preoperative planning to measure the angle, intraoperative implantation according to the planned angle, in order to ensure accurate screw placement. For elderly patients with osteoporosis, care should be taken to avoid spinous process fractures caused by rigid connections between the SPC and the spinous process.

The direction in which the inserted screw invaded the pedicle wall is clinically relevant. Violations of the caudal pedicle and cephalad endplate could contribute to nerve root irritation and disc degeneration. The SPC guide was a medial pedicle violation protective device for implanting the CBT screw because all K-wires went through the guide tube and connected with the knob set medially above the spinous process, and the hardware restricted the screw trajectory closer to the midline. Both groups showed violations of the lateral pedicle and vertebral cortex in the axial plane, which were more likely to damage paravertebral muscles and vessels.

Both the FH and SPC guide groups showed aggression of the cephalic endplate, lateral and caudal pedicle, and vertebral cortex, probably due to the specific trajectory of the CBT approach. Anterior and lateral penetration of the vertebral body can damage paravertebral muscles and blood vessels. In this study, the accuracy rate of the CBT screw placement with the SPC guide was 92.2%, which was significantly higher than the results reported by Kasukawa et al.^20^ The protractor on the SPC guide helped maintain the screw implantation at a precise angle, according to the preoperative planning.

### FJV

In previous studies, FJV was one of the risk factors that resulted in symptomatic adjacent segment disease, which can affect long-term clinical outcomes or even necessitate revision surgery^21,22^. Joint deterioration due to FJV is more common in elderly patients with facet joint hyperplasia^23^. Therefore, it is particularly important to reduce the incidence of FJV to prevent the occurrence of adjacent segment degeneration. Due to the SPC guide being anchored close to the caudal side, the rates of FJV were lower in the SPC guide group than in the FH group. However, the development of adjacent segment degeneration requires longer follow-up, and further studies are therefore warranted.

### Screw angle

The trajectory of the cortical screw was directed approximately 25° cranially and 10° laterally^17^. The cranial angle of the cortical screw trajectory was higher in the SPC guide group than in the FH group (27.7 ± 5.5° vs. 23.0 ± 4.4°, *P* = 0.011). A possible explanation is that the SPC device-guided screw was much more caudal, so a large cranial angle was needed to purchase the cortical bone from the lower starting point. The lateral angle of the cortical screw trajectory was higher in the SPC guide group than in the FH group (10.1 ± 2.0° vs. 6.4 ± 3.1°, *P* > 0.001). This may be because the hardware-restricted screw trajectory was closer to the midline.

### Radiation exposure

No studies have compared the radiation exposure between SPC guides and fluoroscopy-assisted FH techniques. In our study, the radiation dose and duration for both the staff and patients were the same in both groups. This may be because the SPC guide surgical procedure was like FH, and there was no need for additional positioning fluoroscopy compared with FH screw placement. In previous studies^13^, using robotic-assisted screw placement resulted in a reduction in radiation exposure to the medical team, but intraoperative CT scans increased the radiation dose to the patient.

### Operative time and blood loss

The operative time was slightly longer in the SPC guide group than in the FH group. The SPC guide requires a few minutes to insert each screw due to the precise adjustment of the cranial and lateral angles of the guide. The intraoperative blood loss was the same in both groups—no additional or extended incisions were required for the spinal surgery with an SPC guide; this protects the cephalic facet joints from excessive dissection.

### Clinical outcomes

Patients in both groups were similar in terms of clinical recovery. There were significant differences in ODI and back pain VAS scores between preoperative and last postoperative follow-up. Patients who underwent both procedures showed significant improvements in pain intensity and functional status postoperatively compared to preoperatively. At the same time, there was no statistically significant difference in ODI and back pain VAS scores at the last follow-up between the two groups. SPC assisted CBT screw placement was similar to Free-Hand CBT screw placement in improving patient symptoms and clinical outcomes. This is similar to the conclusions of previous cadaveric studies of this device. In cadaver studies^24,25^, our center found that the use of SPC improved screw placement accuracy. Accurate screw placement greatly increases the likelihood of patient symptom improvement and surgical success after surgery.

### Study limitations

This study had some limitations. First, this study was performed on patients who underwent L4/5 single-level spinal surgery, implying that the conclusions may not apply to subjects undergoing other segment and multi-level fusion surgeries. Second, the small sample size limits the evaluation of screw accuracy and surgical radiation exposure, and the conclusions drawn in this study lack confidence and representativeness. Third, a 1-year follow-up period was not sufficient to truly assess the clinical and radiographic results of the screw placement accuracy. One-year follow-up is insufficient to reflect the impact of low FJV in reducing the incidence of adjacent segment degeneration, and facet joint degeneration requires longer follow-up to observe. Finally, all spinal surgeries were performed by the same surgeon; therefore, the study could not account for changes in technology and experience.

## Conclusions

For lumbar cortical bone trajectory devices, SPC-guided screw placement improves accuracy compared to fluoroscopy-assisted FH technique. Patients receiving SPC-guided assisted screw placement achieved equivalent outcomes in patients receiving fluoroscopy-assisted FH techniques. However, long-term efficacy requires longer follow-up and larger sample size studies.

## Data Availability

The datasets used and analyzed during the current study are available from the corresponding author on reasonable request.
